# Exploring the role of green animation advertising influencing green brand love and green customer citizenship behavior

**DOI:** 10.1186/s40359-023-01050-4

**Published:** 2023-01-25

**Authors:** Yipin Zhang, Yi-Chun Yang

**Affiliations:** 1 Department of Digital Media Arts, Zhejiang University of Technology, Hangzhou, China; 2 Faculty of Business and Management, Beijing Normal University-Hong Kong Baptist University United International College (UIC), Zhuhai, China

**Keywords:** Green animation advertising, Green brand love, Green customer citizenship behavior, Stimulus–organism–response (SOR)

## Abstract

This study applied the theory of stimulus–organism–response to test the role of green animation advertising influencing green brand love and green customer citizenship behavior. We used questionnaire survey and the target are those who having the experience of seeing the Apple’s animation advertisement “Earth Day” in China. Finally there were 516 effective samples gathered for analysis. The result indicated that reliability, attractiveness and informativity are the antecedents of green brand love. Green brand love is positively related to green customer citizenship behavior. In addition, the result confirmed the significant mediating effect of green brand love between reliability, attractiveness, informativity and green customer citizenship behavior. This research was conducted only in Apple’s animation case. Hence, the results may not be generalizable to other contexts. Future research can apply the experimental methods and manipulate different forms of green advertising animation to generalize the findings in this area.

## Introduction

Advertisement is a popular communicative marketing tool to influence consumers’ cognition, attitude, and behavior towards the product or brand [1]. In recent years, to make the ads more appealing, more and more companies use animation in the advertisements [2]. Animation is the process of making the motion image by changing the sequence of static images rapidly, the outcome of this process is a motion picture [3, 4]. These animated advertisements give consumers a natural feeling about the brand [5, 6]. The advancement of the new media has also facilitated the development of the animation application [7, 8].

In recent years, improving environmental protection has become an important issue [9]. Due to the increasing number of eco-friendly customers, companies are forced to pay attention to environmental protection activities [10], and many start to focus on eco-friendly products and advertising to fulfill the needs of consumers [11]. In this context, green advertising has rapidly expanded [12]. Green advertising has positive results for business because it can deliver eco-friendly information to consumers [13], hence shaping consumers’ orientation toward sustainable consumption [14]. With the growing role of green advertising, some research are conducted. For example, Agarwal and Kumar [15] proposed a comprehensive overview of the current status of research in green advertising. The study is the only review on the topic of green advertising. Shen et al. [16] confirmed the important roles of advertising creativity on green advertisements.

Despite the related studies in this area, research on the attributes of green advertising has been relatively insufficient. The research objective is to understand the attributes of green animation advertising influencing customers’ attitudes and behaviors, which may be beneficial for companies to conduct suitable marketing strategy in this area. To fill this gap, this research used the theory of stimulus–organism–response to test the attributes of green animation advertising influencing green brand love (attitude) and green customer citizenship behavior (behavior). This theory suggests that external stimulus are signals that influence a person’s internal evaluation and then produces a response [10], which is suitable for explaining consumers’ attitudes and behaviors when responding to the exposing environmental cues [17]. Therefore, we explored the attributes of green animation advertising on green brand love and green customer citizenship behavior. In this study the participants are those who having the experience of seeing the Apple’s animation advertisement “Earth Day” in China. Apple is a typical case because Apple cares for environmental protection and launches a series of green animation advertisements in recent years.

## Literature review and hypothesis

Green advertising refers to advertisement stressing eco-friendly and environmentally responsible behavior [18]. The effect of green advertising can generate consumers’ attitudes and behaviors in related activities [19]. Woohyuk and Seunghee [20] proposed that the attributes of green advertising comprise the three notions, reliability, attractiveness and informativity. Specifically, reliability refers to the attribute in advertising that consumers can trust it [21], and the credibility of advertising can strongly influence customers’ reaction to advertisements and the advertised brands [20]. Attractiveness means the design attractiveness and creativity of the advertising can draw consumers’ attention and strengthen their attitude and responses to eco-friendly advertisements [22]. Informativity means that customers can access relevant information from a detailed description of the advertising product [23]. The power of advertising rests in providing information to maximize consumers’ satisfaction with purchasing products [20]. Extending the concept of green advertising in animation context, green animation advertising refers to the animation advertising stressing eco-friendly and natural issues. Therefore, in this study the concept of green animation advertising can be addressed from reliability, attractiveness and informativity. In this study, reliability means that consumers can trust the attribute in animation advertising. Attractiveness refers to the design attractiveness of the animation advertising to draw consumers’ attention. Informativity means that customers can gather related information from a detailed description of the animation advertising.

Brand love is a type of relationship that consumers establish with brands, encompassing “multiple interrelated cognitive, affective, and behavioral elements, rather than a specific, single, transient love emotion” [24]. When customers generate brand love toward the brand, they view the loving brand as part of their identity [25]. Green brand love means that customers can create emotional connections with a particular brand stressing green activities [26].

Customer citizenship behavior refers to customers’ self-willingness to engage in behavior beneficial for a company [27]. Green customer citizenship behavior means customers’ voluntary involvement in green activities associated with a company’s brand [10]. That is, those who have high level of green voluntary behaviors have the willingness to actively participate in the environmental activities of the company [28].

Due to the imperfect and asymmetrical information, consumers usually rely on signals from the company’s activities to make inferences about the quality of a specific seller [29]. Animation advertising can be a cue for consumers to create confidence about the brand, hence generating brand love [30]. Applying this nature in a green setting, when a brand can deliver the green concern via animation advertising, it may contribute to brand love for the environmentally-friendly brand. Based on the above statement, this study proposes the following hypothesis:

### **Hypothesis 1**

Green animation advertising is positively connected to green brand love.

From a customer’s point of view, loving a brand is a wonderful experience and this makes a customer keep a good relationship with the brand [25]. Brand love enables a customer to exhibit a positive behavior to maintain the loving relationship [24]. Extending the nature of the concept in green context, this study suggests that green brand love is a positive brand involvement in green activities, and this helps a customer to generate the altruistic behavior which assist the environmental brand. Those customers who love a particular brand tend to perform positive behaviors to maintain a loving relationship [31]. Hence, we propose the hypothesis:

### **Hypothesis 2**

Green brand love is positively connected to green customer citizenship behavior.

The noted theory of stimulus–organism–response (SOR) suggests that environment is a stimulus that will cause an individual’s internal evaluation and then produces a response [10]. This theory proposes that an individual’s emotion becomes a crucial role in responding to the exposing environmental stimulus [10]. Deng and Yang [10] used the concept of stimulus–organism–response to confirm the role of green attributes enhancing customers’ feelings and citizenship behaviors. Zhang et al. [32] found that if companies deliver green attributes to customers, it can facilitate customers’ green brand love and green citizenship behaviors. Based on the relate studies, this research suggests that green animation advertising is an external stimulus, which may cause an internal evaluation (green brand love) and then create a response (green customer citizenship behavior). Hence, the following hypothesis is proposed:

### **Hypothesis 3**

Green brand love has a mediating role in the relationship between green animation advertising and green customer citizenship behavior.

### **Hypothesis 3a**

Reliability has a mediating role in the relationship between green animation advertising and green customer citizenship behavior.

### **Hypothesis 3b**

Attractiveness has a mediating role in the relationship between green animation advertising and green customer citizenship behavior.

### **Hypothesis 3c**

Informativity has a mediating role in the relationship between green animation advertising and green customer citizenship behavior.

## Method

### Participants

The target are those who having the experience of seeing the Apple’s animation advertisement “Earth Day” in China. In order to celebrate Earth Day, Apple launched a series of animation advertisements for Earth Day. It is obvious from the advertisements that Apple advocates environmental protection and implies the importance of recycling process for environmental protection issues.

This study used convenience sampling, and before the survey, we confirmed all of the participants seeing the advertisement before. Apple always emphasizes its environmental protection concept to the public, and corporate promotion of environmental protection and social responsibility is indeed a way to enhance its brand image. For example, as part of Apple's ongoing commitment to reducing the environmental impact of its products through innovation, Apple has helped facilitate the development of this technology. Aluminum is almost 100% recyclable and has a better overall environmental footprint than plastic. A new initiative launched by Apple promises to build its devices from 100% recycled materials in the future. In addition, Apple's entire product packaging has reached a 99% recycling rate. Apple's "green marketing" not only introduces environmental protection concepts, but also strives to showcase products and technologies. For example, in the animation "Why Apple Produces Artificial Sweat", in order to ensure the safety and environmental protection of product materials, Apple will create "artificial sweat" in the laboratory, and then put it in the laboratory. The Apple Watch strap is dipped into a bottle of sweat to monitor for toxic spills. The point of this animation is to achieve zero waste, which is consistent with Apple’s green image.

Finally there were 516 effective samples gathered for analysis. Among the 516 respondents, 69.95% were men and 30.05% were women. In addition, approximately 50% of the respondents were aged between 21 and 30 years. Education levels were fairly high, with over 79% having been educated at college level. The majority (62%) of the respondents was not married. Among the respondents, 42% reported a personal monthly income of between CNY$4,000 to CNY$5,000.

### Procedure

This research collected data from Guangzhou of China, we recruited participants via convenience sampling and distributed a total of 600 online questionnaires. We distributed the online questionnaire and the content of Apple’s animation advertisement “Earth Day” in China, and we also explain the meaning of this research. Data collection was conducted between 10 February 2022 and 15 April 2022. All participants are asked to read the content of Apple’s “Earth Day” first, then after that they start to do the survey. In the process they know the purpose, benefits and risks behind the study, and they do not sign and informed consent. The chosen respondents were assured of anonymity and confidential treatment of the answers. All 516 returned questionnaires were subjected to empirical analysis. In addition, we offered all participants a $2 gift certificate to encourage the participants to join the survey.

### Measures

Five constructs were measured in the questionnaire. Respondents answered the items using a five-point Likert-type scale (1 = ‘strongly disagree’; 5 = ‘strongly agree’). The five constructs in the questionnaire included reliability, attractiveness, informativity, green brand love, and green brand attachment. All the items were translated to Chinese version and adjusted to the context of the study. Reliability, attractiveness and informativity were measured based on 12 items from previous study [20], all items were modified to suit the content (animation) of this study (Table 1). For example, the original item such as “I think the green advertising is generally reliable”, we modified the item to “I think the green animation advertising is generally reliable”. Green brand love was measured using four items based on previous study [33]. Sample item such as “I love this green brand”. Green customer citizenship behavior was measured by six items used in previous literature [34]. Sample item such as “I am willing to recommend the environmentally friendly Apple company to others”. Before the formal survey, this study conducts pretest with 50 participants to confirm that the scales were suitable for use.

**Table 1:** Confirmatory factor analysis of green animation advertising items

Items	Factors		
	1	2	3
*Reliability*			
1. I think the green animation advertising is generally reliable	0.72		
2. I trust the information on green animation advertising	0.69		
3. I think the green animation advertising is sincere	0.67		
4. I think the green animation advertising expresses the true nature	0.64		
*Attractiveness*			
1. I think the green animation advertising is interesting		0.71	
2. I think the green animation advertising is novel		0.72	
3. I think the green animation advertising is attractive		0.68	
4. I like the green animation advertisement		0.69	
5. The green animation advertisement catch my attention		0.71	
*Informativity*			
1. I think the green animation advertising provides information on eco-friendliness			0.69
2. I think the green animation advertising is easy to understand			0.71
3. I think the green animation advertisements give me the information I need			0.72
Alpha coefficient	0.93	0.92	0.93
GFI = 0.93 RMSR = .05			

### Data analysis

Using software LISREL VIII, we ran a SEM analysis to examine the model [35]. Confirmatory factor analysis (CFA) was performed as the primary data analysis tool. Results (Table 1) suggested a three-factor structure with an adjusted goodness-of-fit index (GFI) of 0.93 and a root mean square residual (RMSR) of 0.05.

The Alpha coefficient of reliability, attractiveness, informativity, green brand love and green customer citizenship behavior are 0.93, 0.92, 0.93, 0.89 and 0.91. All the constructs are higher than the standard of 0.6 [36], suggesting excellent reliability. Factor loading appears higher than 0.5 suggesting suitable discriminant validity [36]. Convergent validity is also confirmed when the average variance extracted is greater than 0.5 for each construct and the square root of AVE is significantly higher than the correlations between constructs (Table 2).

**Table 2:** Descriptive statistics and correlations among indicator variables

Variables	*M*	*SD*	*AVE*	(1)	(2)	(3)	(4)	(5)
Reliability (1)	4.16	0.71	0.66	**0.806**				
Attractiveness (2)	4.19	0.62	0.61	0.396**	**0.784**			
Informativity (3)	4.23	0.68	0.59	0.402**	0.426**	**0.772**		
Green brand love (4)	4.15	0.82	0.61	0.412**	0.399**	0.411**	**0.779**	
Green customer citizenship behavior (5)	4.16	0.97	0.62	0.406**	0.409**	0.389**	0.419**	**0.748**

n = 516. Figures along the diagonal (in bold) are the values for the square root of the AVE

**p < 0.01

In addition, we can observe that the three constructs of green animation advertising all have positive associations with green brand love and green customer citizenship behavior (Table 2).

## Results

**Fig. 1:**
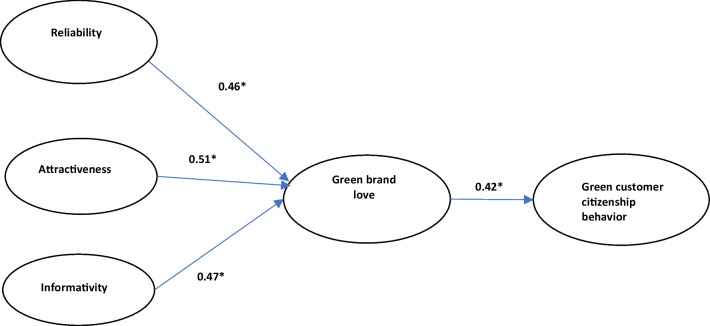
Conceptual model. *Notes*: Standardized path coefficients are reported; *p < 0.05

After running SEM, results show showed goodness-of-fit indices (*chi-square value/d.f.* = 2.204), *RMSEA* = 0.072; *CFI* = 0.92; *IFI* = 0.92; *NFI* = 0.91). The results of the overall fit measures suggested that the model (Fig. 1) fits the data well.

### Hypothesis testing

The results (Table 3) pointed out that reliability, attractiveness and informativity are the antecedents of green brand love (H1a: *coefficient* = 0.46, *p* < 0.05; H1b: *coefficient* = 0.51, *p* < 0.05; H1c: *coefficient* = 0.47, *p* < 0.05). In addition, we test the role of green animation advertising influencing green brand love by using all items including reliability, attractiveness and informativity, the result supports our hypothesis (H1: *coefficient* = 0.48, *p* < 0.05). *H4* posited that green brand love is positively related to green customer citizenship behavior. The result also gets support (*coefficient* = 0.42, *p* < 0.05).

**Table 3:** Results of hypotheses and model statistics

	Path coefficient	Results
Reliability → Green brand love (*H1a*)	0.46*	Supported
Attractiveness → Green brand love (*H1b*)	0.51*	Supported
Informativity → Green brand love (*H1c*)	0.47*	Supported
Green brand love → Green customer citizenship behavior (*H2*)	0.42*	Supported

*p < 0.05

To examine the mediating role of green brand love, Sobel test is used to examine the effect [36]. The result confirmed the significant mediating effect of green brand love between reliability (*p* < 0.05), attractiveness (*p* < 0.05), informativity (*p* < 0.05) and green customer citizenship behavior (Table 4).

**Table 4:** Sobel test’s table

Path	Sobel test
Reliability → green brand love → green customer citizenship behavior	4.82*
Attractiveness → green brand love → green customer citizenship behavior	3.98*
Informativity → green brand love → green customer citizenship behavior	4.36*

*p < 0.05

## Discussion

In this research, we explored how the green animation advertising affect green brand love and green customer citizenship behavior. We found that three attributes of green animation advertising—reliability, attractiveness, and informativity—have the positive associations with green brand love. Moreover, this study shows clearly that green brand love is the determinant of green customer citizenship behavior. And we also confirmed the mediating role of green brand love between green animation advertising and green brand citizenship behavior.

The positive associations between three dimensions of green animation and green brand love confirm the results of Cristela et al. [30] that animation advertising can be a signal for consumers to generate confidence about the brand, hence creating brand love [30]. This study extends the application of animation advertising in green context, and confirms the association between green animation advertising and green brand love. Animation advertising should stress the issue of green in terms of the credibility of advertising, design attractiveness and eco-friendly information, so that consumers can love the green brand.

In addition, prior research suggested that those customers loving the brand tend to perform some positive behaviors to maintain a loving relationship, such as brand loyalty [24]. This loving connection is beneficial to customers’ voluntary behaviors related to environmental activities [31]. Our work furthers the research by confirming the role of brand love influencing customer citizenship behavior in green setting. Those who love the environmentally-friendly brand tend to perform voluntary behaviors in assisting the brand’s activities. This research contributed to the role of green customer citizenship behavior as an outcome of green brand love in the green animation advertising context.

Finally, this research applied the theory of SOR to examine the stimuli of animation advertising in generating brand love and customer citizenship behavior in green context. The finding is congruent with the study of Hong et al. [37] that animation advertising can enhance consumers’ feelings and behaviors. We further confirm the role of green animation advertising influencing consumers’ attitudes and behaviors. The result helps us understand the role of green animation advertising influencing customers’ attitude and behavior, and this enables companies to conduct suitable marketing strategy in animation activities.

This study advances our knowledge via the following contributions. First, the research extends the understanding about how the attributes of green animation advertising influencing customers’ attitude and behavior. Second, the study examines the possible relationships between green animation advertising, green brand love and green customer citizenship behavior in China’s setting. What’s more, with the growing role of brand love [38], some theories were used to capture the concept of brand love [39], such as social exchange theory [40], social identity theory [17] and attachment theory [41]. This research enriches the area by using the theory of stimulus–organism–response (SOR) to explore factors influencing brand love in green context.

### Theoretical contributions

From a theoretical perspective, first, our study fills the research gap by uncovering the role of green animation advertising influence consumers’ branding attitudes and behaviors. We made the significant departure of identifying three attributes (reliability, attractiveness, informativity) of green animation advertising that affect green brand love and green customer citizenship behavior. The related research in green animation advertising is relatively insufficient, with the growing role of this issue, this study provides a base to understand more about the application of green animation advertising in customers’ psychology and behaviors. Second, by confirming the mediating role of green brand love between green animation advertising and green customer citizenship behavior, the result enables researchers to expand SOR theory related to green animation advertising in the marketing and advertising industry.

### Practical implications

In practical management, it is necessary to stress the attributes of reliability, attractiveness, and information when developing green animation advertising strategies seeking to influence consumers’ attitudes and behaviors. First, the marketers can stress the green attribute in advertising to make consumers trust the advertisement. The reliability of advertising can strongly influence an audience’s reaction to advertisements and the advertised brands [42]. Second, to direct consumers to love the eco-friendly brand and perform some positive behaviors, advertisements need to attract the attention of consumers and spark their interest with attractive environmental animation advertising elements. Third, it is also necessary to produce advertisements with environmental messages that draw consumers’ attentions.

### Limitations

This study has some limitations. First, customers’ evaluation of the green animation advertising could differ based on culture. How culture affects customers’ perception of green animation advertising needs further investigation. Second, this research was conducted only in Apple’s animation case. Hence, the results may not be generalizable to other contexts. Third, this study only tested the relationship between specific constructs. Maybe more variables can be added to enrich the research area. Finally, most respondents are concentrated in the age of 20–30, future study can gather more samples to generalize the findings.

### Directions for future research

This investigation is based on the data from China’s consumers. When exploring causal relationships, future longitudinal data can be used to comprehend causality issues. Moreover, future research could examine additional antecedents of brand love in the green context. Future research could consider other commercial activities that might enhance customers’ green brand love and green customer citizenship behavior. What’s more, future research can apply the experimental methods and manipulate different forms of green advertising animation to generalize the findings in this area.

## Data Availability

The datasets used and analysed during the current study available from the corresponding author on reasonable request.
